# A customized scaffolds approach for the detection and phasing of complex variants by next-generation sequencing

**DOI:** 10.1038/s41598-020-71471-3

**Published:** 2020-09-14

**Authors:** Qiandong Zeng, Natalia T. Leach, Zhaoqing Zhou, Hui Zhu, Jean A. Smith, Lynne S. Rosenblum, Angela Kenyon, Ruth A. Heim, Marcia Eisenberg, Stanley Letovsky, Patricia M. Okamoto

**Affiliations:** 1 Integrated Genetics, Laboratory Corporation of America Holdings, 3400 Computer Dr., Westborough, MA USA; 2 Center for Molecular Biology and Pathology, Laboratory Corporation of America Holdings, 1912 T.W. Alexander Dr., Research Triangle Park, Durham, NC USA

**Keywords:** Genetic testing, Next-generation sequencing

## Abstract

Next-generation sequencing (NGS) is widely used in genetic testing for the highly sensitive detection of single nucleotide changes and small insertions or deletions. However, detection and phasing of structural variants, especially in repetitive or homologous regions, can be problematic due to uneven read coverage or genome reference bias, resulting in false calls. To circumvent this challenge, a computational approach utilizing customized scaffolds as supplementary reference sequences for read alignment was developed, and its effectiveness demonstrated with two *CBS* gene variants: NM_000071.2:c.833T>C and NM_000071.2:c.[833T>C; 844_845ins68]. Variant c.833T>C is a known causative mutation for homocystinuria, but is not pathogenic when in *cis* with the insertion, c.844_845ins68, because of alternative splicing. Using simulated reads, the custom scaffolds method resolved all possible combinations with 100% accuracy and, based on > 60,000 clinical specimens, exceeded the performance of current approaches that only align reads to GRCh37/hg19 for the detection of c.833T>C alone or in *cis* with c.844_845ins68. Furthermore, analysis of two 1000 Genomes Project trios revealed that the c.[833T>C; 844_845ins68] complex variant had previously been undetected in these datasets, likely due to the alignment method used. This approach can be configured for existing workflows to detect other challenging and potentially underrepresented variants, thereby augmenting accurate variant calling in clinical NGS testing.

## Introduction

Next-generation sequencing (NGS) refers to a process of massively parallel sequencing that produces a large number of target sequence reads. This technology has been widely used in the genetic diagnostics field as it enables simultaneous and rapid assessment of many clinically relevant genomic regions in patient samples. Variant identification is generally done by aligning sequence reads to a genomic reference in order to assess the presence of any sequence variations^1^, and its analytical sensitivity in variant detection depends greatly on read mapping accuracy. NGS variant detection using short reads (e.g., Illumina sequencing) works well for point mutations, small insertions and deletions (indels), but has limitations in the detection of structural variants and variants residing in repetitive or highly homologous sequences^2^. Even with the most recent human genome builds, GRCh37/hg19 and GRCh38/hg38, which include some alternative loci for highly divergent regions, most aligners are constrained to using one primary sequence as reference. As a result, sample reads that vary greatly from the reference may map either incorrectly or not at all. This reference bias affects the sensitivity and specificity of variant detection, resulting in false positive and false negative calls^3–5^.

Variant phasing is used to identify whether two variants are located within the same copy of a gene (*cis*) or in two different copies (*trans*). Phasing information is imperative for predicting clinical consequences, especially in autosomal recessive disorders. Approaches that statistically infer phasing; i.e., population- or pedigree-based methods, require large cohorts or availability of generational family members for testing, and could be inapplicable to rare or de novo mutations^6,7^. With NGS, algorithms for read-based phasing have been developed to computationally assemble overlapping reads with two or more heterozygous variants into larger haplotype blocks^8–10^. NGS platforms for long-read sequencing can link multiple variants in one sequence read, thereby providing direct phasing information^11^; however, the high cost, low throughput and high per-base error rate have restricted their use in clinical genetic testing. Linked-read sequencing can computationally build and phase long haplotypes at low error rate, but may have limited sensitivity in the detection of small structural variants and has mainly been used in genome phasing^12,13^. In cases of structural variants, genome reference bias can further confound the phasing of even closely linked alleles due to faulty mapping.

To enable the detection and phasing of targeted structural variants using short reads, we developed a computational method that utilizes variant-specific customized reference sequences as scaffolds for read alignment. This customization allows reads from different haplotypes to map correctly onto the corresponding reference scaffolds, resulting in improved genotype determination. A strategy of using custom reference sequences for variant detection has been employed before, but mainly for the assessment of oncogenic gene fusions in cancer^14,15^. Our reference scaffold is tailored to a complex structural variant, where variant phasing is further facilitated by the introduction of a marker in the scaffold sequence that enhances the efficient triaging of reads. These scaffolds can be constructed for any target region and incorporated into an existing variant analysis workflow without extensive algorithm development or increased analysis time.

Feasibility of the custom scaffolds approach was assessed for the detection of a clinically significant, complex variant of the *CBS* gene. The *CBS* gene encodes the enzyme cystathionine beta-synthase, and its deficiency causes homocystinuria (OMIM 236200)^16^. Carrier testing panels may assess pathogenic *CBS* variants, including the single nucleotide variant NM_000071.2:c.833T>C (rs5742905, NP_000062.1:p.Ile278Thr, GRCh37/hg19 chr21:44483184A>G)^17^. This variant has a reported population carrier frequency that ranges from 0.02% (1000 Genomes Project)^18^ to 0.15% (Genome Aggregation Database or gnomAD)^19^ based on chromosome counts. The c.833 T>C variant often occurs in *cis* with a 68 bp insertion, NM_000071.2:c.844_845ins68^20^, to form a benign complex variant, NM_000071.2:c.[833T>C;844_845ins68]. The population carrier frequency for c.[833T>C;844_845ins68] varies greatly among different ethnic groups, ranging from 40–50% in Africans and Northern Europeans to 2.5% in East Asians, and < 1% in Native American populations^16,21^. To date, c.844_845ins68 has been reported to segregate only in *cis* with the c.833T>C mutation; however, several other scenarios are also plausible, including c.[833T>C;844_845ins68] in *trans* with c.833T>C, a complex variant which may even attenuate disease severity^20^. Because clinical interpretation of c.833T>C depends on the presence of and phasing with c.844_845ins68, it is essential that both variants are correctly genotyped and phased in genetic testing.

In the present study, we describe the design of variant-specific custom scaffolds that can correctly differentiate all possible genotype and phasing permutations of the *CBS* variants c.833T>C and c.844_845ins68. We also demonstrate the usefulness of our approach in clinical testing by applying it to the detection of the c.833T>C variant, either alone or as a complex variant c.[833T>C;844_845ins68], in more than 60,000 patient specimens and two 1000 Genomes Project cell line trios. When compared with the conventional method of using one primary sequence as reference for alignment, the customized scaffolds method not only circumvents issues with mapping bias, but also yields important phasing information, thereby improving detection of complex variants that may be underestimated in population datasets.

## Results

### Custom scaffolds composition and variant call analysis

**Figure 1:**
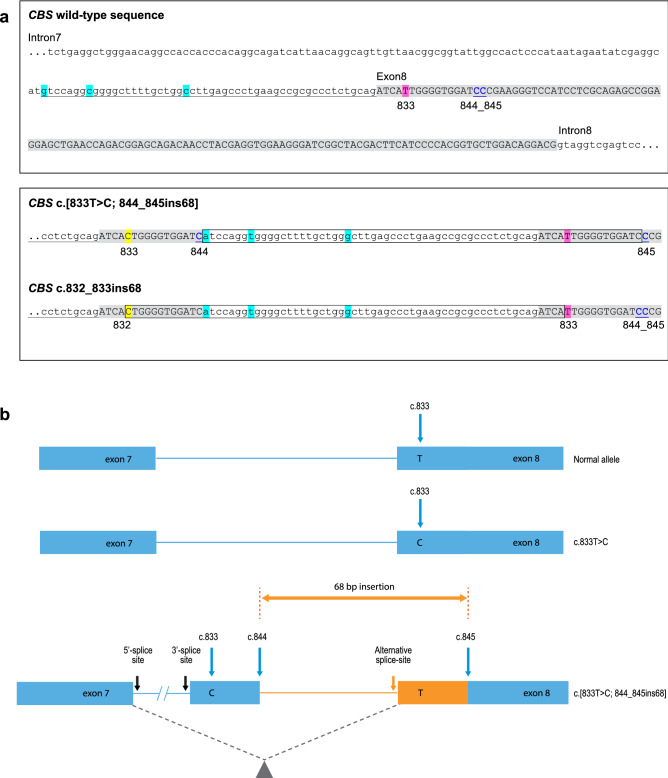
***CBS*** **c.833T>C and the complex variant, c.[833T>C;844_845ins68] on chromosome 21.** (**a**) Sequence structures of the 68 bp insertion in *cis* with c.833T>C show that the variant descriptions for c.[833T>C;844_845ins68] and c.832_833ins68 in ClinVar and gnomAD are equivalent. In both panels, coding exon 8 is shaded in gray with the c.833 wild-type base highlighted in pink and the locations of the bases that differ between the wild-type and 68 bp insertion sequences in blue. The duplicated intronic sequence is underlined. In the lower panel, the 68 bp insertion sequences are shown in boxes with the relative locations of the c.833C variant in *cis* highlighted in yellow. Intronic sequence is in lowercase. (**b**) Schematic representation of the variant region depicts the exons in blue with the locations of the c.833 base as a normal [T] or pathogenic [C] variant, and the 68 bp insertion between the c.844 and c.845 bases in orange. Dotted lines in gray indicate the region that is excised due to alternative splicing.

To define the 68 bp insertion sequence for use in our custom scaffolds approach, we searched the gnomAD^19^ and ClinVar^22^ databases and found that there were two descriptions for the variant: NM_000071.2:c.844_845ins68 (ClinVar Variation ID 212823) and NM_000071.2:c.832_833ins68 (ClinVar Variation ID 226482). These descriptions differed in their insertion sites and sequences, and when c.833T>C was present in *cis*, its location could be assigned as either part of the insertion sequence (i.e., c.832_833ins68) or separate from it (i.e., [c.833T>C; 844_845ins68]). Upon further scrutiny, we concluded that the two descriptions were equivalent since the sequences for the 68 bp insertion and wild-type genome are identical at the sites in which the insertions occur in the exon (Fig. 1a). In order to distinguish c.833T>C from the insertion sequence in our analysis as well as for consistency, we refer to the 68 bp insertion as c.844_845ins68 and the *cis* variant as c.[833T>C; 844_845ins68] throughout this paper.

The c.844_845ins68 variant occurs near the 5′-end of coding exon 8 of the *CBS* gene and, with the exception of a few divergent bases, is a duplication of the last 52 bases of intron 7 and the first 16 bases of exon 8 (GRCh37/hg19 chr21:44,483,173–44,483,240). The inserted sequence includes a copy of the wild-type base, c.833T, as well as the 3′-splice site at the intron7-exon8 junction, the latter of which causes alternative splicing in the transcript. As a result, the c.833T wild-type base in the insertion is retained while the c.833T>C variant is excised if it is present in *cis* with the insertion^23–26^ (Fig. 1b). We also note that two alternate alleles of the c.844_845ins68 variant have been reported in gnomAD (v2.1.1). One allele differs by 3 bases from the GRCh37/hg19 reference genome (~ 96% identical; hereafter referred to as the common 68 bp insertion) and is more prevalent in the general population than the other, which has an additional divergent base (~ 94% identical with hg19) and is identified only in 16 individuals of African ethnicity (denoted hereafter as the rare 68 bp insertion). Nevertheless, with either allele sequence, the high similarity between the 68 bp insertion and the reference genome sequences (e.g., GRCh37/hg19 or GRCh38/hg38) causes forced alignment of reads having the c.[833T>C;844_845ins68] complex variant to a standard genome assembly, thereby complicating detection of the insertion and/or the c.833T>C variant and potentially leading to a false call.

**Figure 2:**
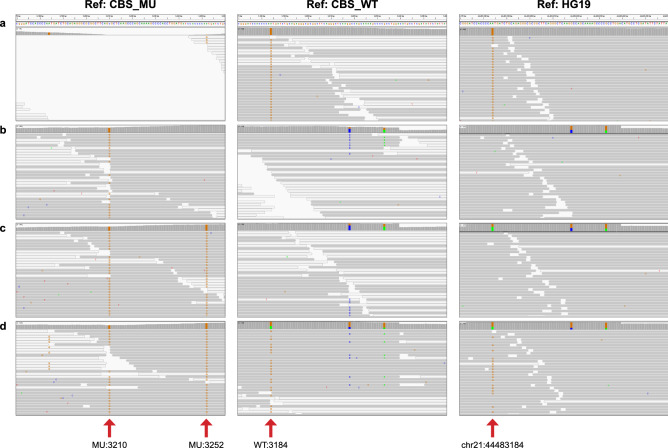
**Alignment of simulated reads to the custom scaffolds and hg19 reference genome.** Examples of simulated read alignments to custom scaffolds CBS_MU (left panel) and CBS_WT (middle panel) and to GRCh37/hg19 reference genome (right panel) are visualized using the Integrative Genomics Viewer (IGV; Broad Institute, Cambridge, MA). Arrows point to the locations of the informative bases that are used for variant calling on each scaffold and the hg19 reference. (**a**) c.833T>C detected at WT:3184 on Ref:CBS_WT or chr21:44,483,184 on Ref:HG19; (**b**) c.844_845ins68 detected with Ref:CBS_MU at MU:3210G>C; (**c**) c.[833T>C;844_845ins68] detected at both MU:3210G>C and MU:3252A>G on Ref:CBS_MU; and (**d**) c.833T>C in *trans* with c.[833T>C; 844_845ins68] detected at MU:3210G>C and MU:3252A>G as HOM on Ref:CBS_MU and at WT:3184A>G as HET on Ref:CBS_WT. For (**a**–**c**), alignments are shown for simulated homozygous samples. For (**b**–**d**), a minority of reads with the 68 bp insertion will align to Ref:CBS_WT and Ref:HG19 due to high sequence homology as shown in the per-base tracks for two of the three mismatched bases (blue “C”, green “A”). A third divergent base is not visible because of soft-clipping by the variant caller.

To address this challenge, we constructed two custom scaffolds (CBS_WT and CBS_MU) to discriminate reads with and without the 68 bp insertion (Fig. 2). CBS_WT scaffold represents the normal genotype and encompasses 5000 bp of the *CBS* gene region at chr21:44,480,001–44,485,000 based on GRCh37/hg19. CBS_MU scaffold is the insertion genotype and consists of the same genomic region as the CBS_WT scaffold plus the common 68 bp insertion sequence. Due to the mismatch and gap penalty cost differences in the alignment algorithm, only reads that have the insertion sequence will align to the CBS_MU scaffold. To facilitate efficient variant calling and phasing, the CBS_MU scaffold was also designed with an additional G>C base change at scaffold position 3210 (designated CBS_MU:3210). The wild-type base at this position is highly conserved among primates^16^ and has no known polymorphisms or pathogenic variants according to the 1000 Genomes Project and dbSNP150 databases. Alteration of the “G” to a “C” base in the CBS_MU scaffold creates an expected mismatch that serves as a marker for a predetermined variant call to easily identify reads that have the 68 bp insertion (Fig. 2b, Ref:CBS_MU). If the c.833T>C variant also occurs in the same paired-end reads as the 68 bp insertion, it would be detected as a base change at position 3252 in the CBS_MU scaffold (i.e., CBS_MU:3252A>G; Fig. 2c, Ref:CBS_MU). On the other hand, reads with just the c.833 T>C variant will align to the CBS_WT scaffold, and this pathogenic variant will be identified as CBS_WT:3184A>G (Fig. 2a, Ref:CBS_WT).

### Assessment of the customized scaffolds approach using simulated reads

To assess the custom scaffolds for read mapping specificity, simulated reads representing all possible genotype combinations were generated using the ART simulation tool kit^27^. The variant calls for each tested genotype and their corresponding zygosities are compiled in Table 1, which also functions as the lookup table in the genotype calling process, and diagrammed schematically in Supplementary Fig. S1 online.

**Table 1:** **Genotyping and phasing of** ***CBS*** **variants with c.833T>C and/or c.844_845ins68 based on simulated reads.**

*CBS* genotypes	Scaffold alignment		Zygosity calls		
	Ref:CBS_WT	Ref:CBS_MU	WT:3184 A>G	MU:3210 C>G	MU:3252 A>G
c.833T>C	Y	–	HET or HOM	–	–
c.844_845ins68	–	Y	–	HOM	–
c.[833T>C;844_845ins68]	–	Y	–	HOM	HOM
c.[833T>C] in *trans* with c.[844_845ins68]	Y	Y	HET	HOM	–
c.[833T>C] in *trans* with c.[833T>C;844_845ins68]	Y	Y	HET	HOM	HOM
c.[844_845ins68] in *trans* with c.[833T>C;844_845ins68]	–	Y	–	HOM	HET
Wild-type	Y	–	–	–	–

Shown are the custom alignment scaffolds used and the expected zygosity of the informative base for each genotype tested by simulation. A heterozygous call (HET) is determined by an allele frequency between 20% and < 80%, while a homozygous call (HOM) has an allele frequency ≥ 80%.

As expected, reads with just the c.833T>C variant in a simulated homozygous sample aligned only to the CBS_WT scaffold (Fig. 2a, Ref:CBS_WT compared to Ref:CBS_MU). Reads with the c.844_845ins68 variant preferentially aligned to the CBS_MU scaffold (Fig. 2b–d, Ref:CBS_MU) and were easily identified by the homozygous (HOM) G>C readout at CBS_MU:3210, whereas co-occurrence of a HOM base change at CBS_MU:3252A>G, indicated that the 68 bp insertion is present in *cis* with c.833T>C (simulated HOM sample in Fig. 2c, Ref:CBS_MU). We note that because of high homology between the insertion and reference sequences, a fraction of reads with the c.844_845ins68 sequence also aligned to the CBS_WT scaffold due to soft-clipping of the unaligned read ends. The resulting increase in coverage depth in the alignment may suggest that there is a structural variant; however, the 68 bp insertion sequence is not identified as such since the variant caller ignores the soft-clipped bases (simulated HOM sample in Fig. 2b, Ref:CBS_WT). The Ref:CBS_MU scaffold is also used to determine the genotype of a sample with two copies of c.844_845ins68, one that is in *cis* with c.833T>C and the other on the opposite allele (Table 1, last row; Supplementary Fig. S1 online). In this case, base changes at CBS_MU:3210G>C and CBS_MU:3252A>G that are HOM and heterozygous (HET), respectively, would identify the c.844_845ins68 in *trans* with c.[833T>C;844_845ins68]. Alternatively, if c.833T>C occurs in *trans* to c.[833T>C;844_845ins68], read alignments to both the CBS_MU and CBS_WT scaffolds would be observed with CBS_WT:3184A>G, CBS_MU:3210G>C and CBS_MU:3252 A>G calls that are HET, HOM, and HOM, respectively (Table 1, penultimate row; Fig. 2d and Supplementary Fig. S1 online). The change in zygosity for CBS_WT:3184 A>G from HOM to HET is expected due to the presence of c.833T>C on both alleles.

Compared to the custom scaffolds approach, alignment to the GRCh37/hg19 reference genome posed a challenge for detection and phasing of the *CBS* variants (Fig. 2b–d, Ref:HG19). Since the 68 bp insertion is essentially a duplication of the wild-type sequence, all reads with this variant, whether alone or in combination with c.833T>C, were forced to align to the hg19 reference genome by soft-clipping the unaligned read ends at the left and right breakpoints. The resulting increase in read coverage in the alignment may indicate an insertion (Fig. 2b–d, Ref:HG19). However, because of the additional presence of a wild-type c.833T base in the insertion sequence, the c.833T>C allele frequency at chr21:44,483,184 could fall below the variant calling threshold for a HET, thereby impacting detection of the c.833T>C variant, or for a HOM, drop to a frequency in which the zygosity is called incorrectly (Fig. 2c, Ref:HG19). In contrast, reads with only the pathogenic variant c.833T>C readily aligned with the HG19 reference sequence and the variant was easily detected at chr21:44,483,184 (Fig. 2a, Ref:HG19). Alignment of reads with the variant in *trans* with the c.[833T>C;844_845ins68] complex variant had a similar profile to that of c.833T>C by itself but with a lower allele frequency for the mutant base at chr21:44,483,184 (Fig. 2d, Ref:HG19).

### Detection of *CBS* complex variants in two trios by custom scaffolds

To further evaluate performance of the custom scaffolds approach, we compared the *CBS* variant calls to those from single reference genome mapping by analysing NGS data for the 1000 Genomes Project trios, CEPH/UTAH Pedigree NA12878/NA12891/NA12892 and the YRI Pedigree NA19240/NA19239/NA19238^18^. By Sanger sequencing, we had identified and confirmed that the c.833T>C and c.844_845ins68 variants were present in *cis* for NA12892 (CEPH mother), NA19240 (YRI daughter) and NA19238 (YRI mother).

With custom scaffolds as the references, all three 1000 Genomes Project samples were correctly genotyped for the c.833T>C variant in *cis* with the 68 bp insertion as indicated by base changes at CBS_MU:3210 and CBS_MU:3252 (Supplementary Fig. S2 online, Ref:CBS_MU and Ref:CBS_WT). Allele segregation of c.[833T>C;844_845ins68] in both trios corresponded to the expected Mendelian inheritance pattern, thereby confirming correct phase assignment by the custom scaffolds method. However, when the GRCh37/hg19 build was used as the reference, the variant caller struggled to map reads from samples with the c.[833T>C;844_845ins68] complex variant to the reference genome, and only the 68 bp insertion could be detected either directly or inferred as a structural variant in the alignment based on increased read coverage (Supplementary Fig. S2 online, Ref:HG19).

A closer examination of the variant data from the 1000 Genomes Project (NCBI 1000 Genomes Browser Phase 3, ver3.7)^18^ revealed that the c.[833T>C;844_845ins68] complex variant was not called for either NA19240 or NA19238; no *CBS* genotype data for these variants were available for NA12891. The overall population carrier rate for c.833T>C was found to be 0.02% in the 1000 Genomes Project (accessed 11/30/2019 via https://www.ncbi.nlm.nih.gov/projects/SNP/snp_ref.cgi?rs=5742905), and 0.15% in gnomAD (accessed 11/30/2019 via https://gnomad.broadinstitute.org/variant/21-44483184-A-G); information on the frequency for the complex variant in any of the 1000 Genomes datasets was not available. This apparent inconsistency suggests that the c.833T>C variant likely was missed in the two trios when it occurred in *cis* with the 68 bp insertion because of the NGS alignment method and variant calling methods used in the 1000 Genomes Project (i.e., GATK UnifiedGenotyper, FreeBayes and BCFtools)^28^. To further explore the NGS data, we employed a second variant calling method, GATK HaplotypeCaller (ver.4.1.2), which uses local de novo reassembly of reads instead of existing mapping data to simultaneously detect SNPs and indels for any region with variation^29^. Using GRCh37/hg19 as the reference to realign the assembled reads, the GATK HaplotypeCaller also called the *cis* variant in NA19240, NA19238 and NA12891. Our results using the custom scaffolds were in agreement with the GATK HaplotypeCaller, which is also used as the variant caller in gnomAD and considered to be the standard method for variant calling. Along with our simulation study, we further demonstrate with these trio cell lines that the *CBS* variant call accuracy was diminished when calls were made from aligned reads which were mapped directly to the entire genome (Supplementary Fig. S2 online, Ref:HG19).

### Frequency of *CBS* complex variant by population in a cohort received for carrier screening

We applied the customized scaffolds approach in the analysis of 60,318 consecutive specimens from throughout the United States that had been referred to our clinical laboratory for carrier testing of variants in 144 genes associated with more than 115 autosomal recessive and X-linked disorders. The cohort consisted of 89% females (mean age: 30.7 ± 6.0 years) and 11% males (mean age: 35.4 ± 7.0 years). The majority of individuals tested (81%) did not indicate known family history for any of the genetic disorders.

**Figure 3:**
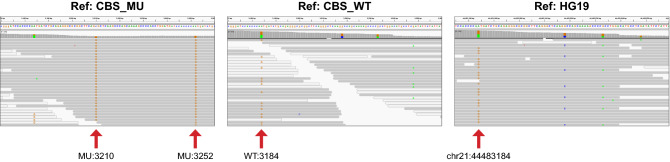
**Detection of the c.833T>C single nucleotide variant in** ***trans*** **with the complex variant, c.[833T>C;844_845ins68].** IGV screenshots display read alignments for a clinical sample in which the pathogenic variant, c.833T>C, is in *trans* with c.[833T>C;844_845ins68] using either the custom scaffolds or GRCh37/hg19 genome as reference. Variant detection utilizes the informative bases on Ref:CBS_MU and Ref:CBS_WT as indicated by arrows at MU:3210 for the 68 bp insertion, MU:3252 for the c.833T>C variant in *cis* with the 68 bp insertion and WT:3184 for the c.833T>C variant on the opposite allele (see also Table 1 and Supplementary Fig. S1 online). Per base coverage tracks show the expected zygosity calls for MU:3210, MU:3252 and WT:3184 as HOM, HOM and HET, respectively. Read alignment to Ref:HG19 shows that only the c.833T>C variant can be detected at chr 21:44,483,184.

As shown in Table 2, a carrier rate of 18.49% was determined for the c.[833T>C;844_845ins68] complex variant and 0.17% for the pathogenic c.833T>C variant with this cohort. Our observed carrier rate for c.[833T>C;844_845ins68] is consistent with the reported range for general population frequencies of up to 40% in African/African Americans and less than 1% in Native American populations^30^. For a small number of clinical cases (0.012%), c.833T>C was also detected as part of the benign complex variant c.[833T>C;844_845ins68] on one chromosome, and as the pathogenic single nucleotide variant c.833T>C on the other (Fig. 3). No incidence of the c.833T>C variant in *trans* with the 68 bp insertion was observed in our specimen set, which is consistent with published population studies^16,23,30,31^.

**Table 2:** ***CBS*** **c.833T>C and c.[833T>C; 844_845ins68] carrier rates.**

Ethnicity	Total number of samples		c.833T>C			c.[833T>C;844_845ins68]		
	gnomAD	Current study	gnomAD, %AF (n)	Current study, %AF (n)	*P*	gnomAD, %AF (n)	Current study, %AF (n)	*P*
All Ethnicities	13,970	60,318	0.17 (24)	0.17 (103)	1	21.10 (2948)	18.49 (11,153)	< 0.0001
African/African American	4359	177	0.05 (2)	0	1	38.72 (1688)	45.2 (80)	0.08
Latin American	424	323	0	0	–	12.54 (53)	16.1 (52)	0.17
Ashkenazi Jewish	145	169	0	0	–	7.93 (11)	10.65 (18)	0.44
East Asian	780	382	0	0	–	0.26 (2)	4.71 (18)	< 0.0001
European	7718	518	0.29 (22)	0.39 (2)	0.66	13.7 (1057)	14.29 (71)	1
Other (e.g., mixed ethnicities)	544	292	0	0	–	14.76 (80)	16.44 (48)	0.55

Comparison of the carrier rates determined in this study v. gnomAD and corresponding *P* values from the Fisher’s Exact test are shown. The gnomAD population frequencies for c.833T>C were calculated using chromosome counts for rs5742905 for all available groups. Variant allele frequencies for the gnomAD European population are from the NFE group and exclude the Finnish group. The c.[833T>C; 844_845ins68] complex variant is described as c.832_833ins68 in gnomAD. Statistical analysis used α = 0.05 as the level of significance for rejecting the null hypothesis. AF, allele frequency; n, number of positive samples; *P*, *P* value.

A subset of 1861 clinical samples with self-reported ethnicity was further analysed to estimate carrier rates for different ethnic groups. As a comparison, the chromosome-based population frequencies for c.833T>C (rs5742905) in gnomAD were extracted and converted to carrier rates. Table 2 shows that the overall c.833T>C carrier rates (All Ethnicities; *P* = 1) and those for individuals of European ancestry were very similar between gnomAD and this study (*P* = 0.66). Except for the East Asian group, the population frequencies for the c.[833T>C;844_845ins68] complex variant among the different ethnic groups analysed in this study were also consistent with frequencies found in gnomAD (Table 2). For the East Asian group, the carrier rate in our clinical specimens was much higher than in gnomAD (*P* < 0.0001), and closer to the carrier rate previously reported in the Chinese population^32^. In addition, 85 samples of various ethnicities were identified as heterozygous for c.833T>C in *cis* with the rare 68 bp insertion sequence using the CBS_MU scaffold. Of these, 14 were compound heterozygotes for the c.[833T>C; 844_845ins68] complex variant and the common 68 bp insertion sequence (Supplementary Fig. S3 online).

## Discussion

The success of NGS in high-throughput sequence variant detection with human genomic DNA has revolutionized the field of clinical genetic testing in the past few years. NGS performance for the detection of single nucleotide variants and small insertion or deletion variants is highly accurate^33,34^ using the current standard human reference genome build for read alignment. However, the single reference genome approach frequently falls short for variant detection in regions with duplications, pseudogenes, paralogous genes or complex variants because reads from different haplotypes are forced to align to the same genomic region, leading to false negative and false positive calls. In this study, we have shown, using two clinically important *CBS* variants as examples, that our customized variant-specific scaffold approach enables reads for these complex regions to segregate by haplotypes. Incorporation of a predefined mismatch at a highly conserved base further customizes the mutant scaffold for efficient triaging and phasing of the variant calls. When applied in combination with standard NGS pipelines that utilize the current human reference genome build, our method can improve the performance of the variant calling process.

The custom scaffold approach has made it possible to determine variant type and obtain phasing information with high sensitivity and accuracy. Using simulated short reads as input, all variant and phasing combinations for the *CBS* complex variant, c.[833T>C;844_845ins68], were successfully resolved, thereby improving sensitivity and specificity of the assay. Our analysis of two widely studied trios from the 1000 Genomes Project revealed the presence of the c.[833T>C;844_845ins68] complex variant in a parent–child duo that segregated as expected for a Mendelian inheritance pattern and was concordant with the gold-standard Sanger sequencing results. Attempts to detect this variant with callers that directly use aligned reads on a standard genome reference were not successful (Supplementary Fig. S2 online). It was also not surprising to find that the variant allele frequency for c.833T>C was underestimated when it occurred in *cis* given that the 68 bp insertion, which also includes the c.833T wild-type base, is almost identical in sequence to the reference genome. Phasing and imputation errors of rare variants in the 1000 Genomes data have been attributed to the limited sample size^35^. Our findings suggest, though, that the c.[833T>C;844_845ins68] complex variant may have remained undetected in the 1000 Genomes samples as a result of the alignment and variant calling methods used in the original NGS analysis, and that there may be other complex or rare variants in these data that also have gone underreported.

Application of the customized scaffolds to a large cohort of anonymized clinical samples (> 60,000 specimens) has provided population risk data for the pathogenic variant c.833T>C and carrier frequency for the benign complex variant c.[833T>C;844_845ins68]. Since these samples were accrued from carrier testing, we cannot rule out ascertainment bias; however, as reported by others^30^, we also observed c.[833T>C;844_845ins68] at a much higher frequency (18.49%) than the pathogenic c.833T>C variant (0.17%) in our sample set. Our observed overall frequency for c.833T>C was approximately the same as in gnomAD (*P* = 1; Table 2). Furthermore, the allele frequency for c.[833T>C; 844_845ins68] has been reported to vary by more than 100-fold depending on the ethnic population^30^. In this study, the overall allele frequencies for c.[833T>C;844_845ins68] at 18.49% and 21.1% were statistically different between our cohort and gnomAD, respectively (*P* < 0.0001). This could reflect differences in the ethnic composition since we do not know the ethnic makeup for most of our cohort. For a small subset of our samples (~ 3.1%) in which ethnicity was voluntarily provided, the allele frequencies were comparable between the two sample sets for all except the East Asian population (0.26% vs. 4.71%; *P* < 0.0001; Table 2), suggesting that the samples for this group may have a different demographic composition in our dataset.

It is also worth noting that although the CBS_MU scaffold was designed with the common 68 bp insertion allele, the c.833T>C variant in *cis* with the rare c.844_845ins68 allele was detected using this scaffold in 0.14% of the samples. All were heterozygous for c.[833T>C;844_845ins68], and of these, 0.02% were compound heterozygous with the c.[833T>C;844_845ins68] complex variant that had the common 68 bp insertion allele, indicating that our approach can identify and phase other variations of the insertion. Within our cohort, we also identified six samples with c.833T>C in *trans* with c.[833T>C;844_845ins68], resulting in a genotype frequency of 0.012%. This compound heterozygosity had been previously identified in a patient affected with a mild form of homocystinuria in an Italian cohort^20^, but until now, and to the best of our knowledge, no genotype frequency information had been reported.

As long as a prior knowledge of the target sequence is available, our custom scaffold alignment method can be easily adapted to detect other technically challenging variants without the need for expensive equipment or extensive customization of the computational method. For example, we have utilized our approach in the detection of a pathogenic 55 bp deletion in the beta-glucosidase gene, *GBA* (OMIM 606463; chr1:155,204,239–155,214,653 in GRCh37/hg19). Due to the gene’s high homology and close proximity to a pseudogene, *GBAP1* (chr1:155,183,616–155,188,809 in GRCh37/hg19), complex recombinant alleles between the gene and pseudogene occur that make it challenging to differentiate these rearrangements from pathogenic variants in *GBA* by NGS^36,37^. Detection of the 55 bp deletion in the *GBA* gene is particularly difficult because the *GBAP1* pseudogene is also naturally missing the analogous sequence. A different NGS scaffold method, which used the entire *GBA* reference gene for alignment, was reported to be unsuccessful at detecting the 55 bp deletion in a recombinant allele that had been enriched by long-range PCR^37^. In contrast, variant calling using our approach, which only required the design of mutant and wild-type scaffolds, detected the true 55 bp deletion in *GBA* as confirmed by Sanger sequencing (manuscript in preparation). We also envision that our method could be applicable to larger variants with known breakpoints such as well-characterized gene fusions and retrotransposon insertions, e.g. the 3 kb insertion founder mutation in the *FKTN* gene that causes a form of congenital muscular dystrophy in the Japanese population^38^.

Assembly-based variant callers such as GATK HaplotypeCaller, albeit limited by the read or insert length, are the standard methods for genotyping and phasing of clinically significant variants, especially those which are less well-known than the *CBS* complex variants that were used as examples in this feasibility study. As mentioned above, the use of our scaffolds approach is restricted to well-characterized variants and a good understanding of the genomic regions in which they reside. Besides applications in clinical testing, for which it was originally developed, this approach can also be useful in clinical genetic research such as in prevalence studies. With the development and optimization of deep learning variant callers like DeepVariant^39^ and Clairvoyante^40^, it may also become possible to use these callers to identify novel or less-known variants in difficult genomic regions. Custom scaffolds with applicable conserved bases could then be designed to augment variant detection for regions that are particularly problematic.

In conclusion, our results show that the custom scaffolds alignment approach can accurately call and phase variants using a standard NGS pipeline and the current human reference genome build without the added cost of specialized instruments, reagents or software. While phasing capability is limited to the read length, this approach is especially suited for the detection and phasing of complex structural variants that occur within challenging intragenic regions, e.g., those that are repetitive or highly homologous to other genes. As we have demonstrated with two clinically important *CBS* variants, the ability to detect and accurately genotype rare and/or complex variants, especially for clinical tests such as carrier testing, will enable better reproductive risk assessment and will help guide physicians in the management of patient care.

## Methods

### Cell lines and specimens

Cell line DNA of two parent–child trios from the 1000 Genomes Project, representing populations of Northern/Western European ancestry in Utah (CEPH/UTAH; NA12878, NA12891, and NA12892) and the Yoruba in Ibadan, Nigeria (YRI; NA19240, NA19239 and NA19238), were purchased from the Coriell Institute for Medical Research (Camden, NJ). De-identified *CBS* genomic data were derived from anonymized clinical specimens that underwent Inheritest Carrier Screen panels testing at our CLIA-certified clinical testing laboratory (Integrated Genetics, Laboratory Corporation of America Holdings, Westborough, MA).

Testing performed was in compliance with the Clinical Laboratory Improvement Amendments of 1988 (CLIA) federal regulations and College of American Pathologists (CAP) accreditation standards. Per the United States Code of Federal Regulations for the Protection of Human Subjects, institutional review board exemption is applicable due to de-identification of the presented data (45 CFR part 46.101(b)(4)). Written informed consent was obtained from all patients by the referring physician at the time that testing was ordered.

### Inheritest Carrier Screen NGS panels

The Inheritest Carrier Screen NGS tests are multigene panels that include the *CBS* gene. Library preparation and target enrichment were performed using the Agilent SureSelectXT method (Agilent Technologies, Santa Clara, CA). Briefly, genomic DNA was fragmented by sonication (Covaris, Woburn, MA), followed by end-repair, A-tailing and adaptor ligation. A target enrichment step was carried out using custom Agilent RNA probes, which were designed to capture all coding exons and flanking sequences of all isoforms for 144 genes (~ 0.5 M bases total), followed by post-capture indexing of the libraries by PCR for multiplex sequencing. Up to 96 libraries were pooled together and paired-end sequenced at 2 × 150 cycles using either the MiSeqV3 chemistry or the HiSeq2500 Rapid Run mode (Illumina, San Diego, CA) at 15 × minimum base coverage. Raw sequencing data were demultiplexed with the Illumina CASAVA v.1.8.2 software to generate fastq sequences for each sample.

### Variant detection

Illumina paired-end sequence reads were used as input for an internally developed and validated variant detection workflow using the CLCbio Genomic Server (version 9.1.1) and Workbench software (version 10.1.1) (Qiagen Bioinformatics, Redwood City, CA). This workflow had two branches. The main branch analysed all sequencing reads for the Inheritest Carrier Screen panels by first trimming the reads to remove library adaptor sequences and low quality bases (Q20 minimum quality threshold), and then aligning them to GRCh37/hg19 human genome build, followed by duplicate reads removal, local realignment, variant detection and annotation. For detection and phasing of the *CBS* variants c.833T>C and c.[833T>C;844_845ins68], a second workflow branch for read alignment to two custom scaffolds was implemented after the read trimming step. The first scaffold, CBS_WT, was identical to the hg19 reference region chr21:44,480,001–44,485,000. The second scaffold, CBS_MU, was also from the same region, but included the common 68 bp insertion plus an introduced G>C base change within the 68 bp insertion that served as a marker to detect the insertion. After read mapping to the scaffolds, duplicate reads were removed prior to variant analysis and phasing determination. The output from this workflow branch was fed into the main branch output to create the final variant report.

Analysis of the two *CBS* variants in the 1000 Genomes trios was also carried out using the HaplotypeCaller in the Genome Analysis Toolkit (GATK ver. 4.1.2; Broad Institute, Cambridge, MA) following the recommended GATK Best Practices workflows^29^, and using GRCh37/hg19 build as the reference sequence. Alignment of simulated reads was visualized using Integrative Genomics Viewer (IGV, Broad Institute, Cambridge, MA)^41^.

### Simulation of variant reads

An in-house Python script was developed to generate simulated variant reads utilizing the ART package^27^, which uses platform-specific error models and base quality profiles for read simulation. The parameters for paired read simulations used in this study were as follows: 200X coverage, 150 bp read length, 300 bp fragment size, 100 bp fragment size standard deviation, and Illumina HiSeq error model. The input sequence template for simulation with the ART tool represented all possible genotype and phasing combinations of the two *CBS* targeted variants, c.833T>C and c.844_845ins68, as well as 500 additional flanking bases on either side of the targeted variants. All genotype combinations of c.833T>C and c.844_845ins68 used in simulation input had been confirmed to report the correct genotype assignment after variant calling.

### Sanger confirmation

*CBS* variants detected by NGS were confirmed by Sanger sequencing with internally designed target-specific, M13-tagged primers:

- Forward: 5′-TGTAAAACGACGGCCAGTCCACCACCCACAGGCAGAT-3’.
- Reverse: 5′-CAGGAAACAGCTATGACCGCGGGGCTTGCCCTTCTGTT-3’.

PCR amplification was performed using 0.25U AmpliTaq Gold DNA polymerase (ThermoFisher Scientific, Waltham, MA) in 1X PCR Buffer II, 1.5 mM MgCl_2_, 50 µM dNTPs, and 250 nM primers and the following cycling condition: 1 cycle at 95 °C for 10 min; 35 cycles of denaturation at 95 °C for 20 s, annealing at 60 °C for 30 s and extension at 72 °C for 60 s; 1 cycle at 72 °C for 3 min. Sanger sequencing was carried out using BigDye Terminator v3.1 Cycle Sequencing kit (ThermoFisher Scientific, Waltham, MA), and capillary electrophoresis on the ABI3730XL (ThermoFisher Scientific, Waltham, MA). SeqScape v2.5 (ThermoFisher Scientific, Waltham, MA) was used for sequence analysis and visualization.

### Statistical analysis

The Fisher’s Exact test was carried out at α = 0.05 significance level using R software ver. 3.6.3. Testing was done using 2 × 2 contingency tables for all ethnicities and for each population.

## Supplementary information

Supplementary Information.

## Data Availability

The clinical dataset generated and analysed during the current study are not publicly available due to privacy considerations imposed by Federal and State laws and regulations; anonymized data are available from the corresponding authors on reasonable request. All other data generated or analysed during this study are included in the published article (and its Supplementary Information files).
