# Supplementary material for: A customized scaffolds approach for the detection and phasing of complex variants by next-generation sequencing
Source: Sci Rep. 2020 Sep 14;10:15060. doi: 10.1038/s41598-020-71471-3 (PMC7490669; doi:10.1038/s41598-020-71471-3)
Supplement: Supplementary file 1 — Supplementary Information. [file 41598_2020_71471_MOESM1_ESM.pdf]

Supplementary Figures

A Customized Scaffolds Approach for the Detection and Phasing of Complex Variants by Next-Generation Sequencing

Qiandong Zeng*, Natalia Leach, Zhaoqing Zhou, Hui Zhu, Jean A. Smith, Lynne S. Rosenblum, Angela Kenyon, Ruth Heim, Marcia Eisenberg, Stanley Letovsky and Patricia Okamoto*

*Correspondence to zengq@labcorp.com or okamotp@labcorp.com

Supplementary Figure S1

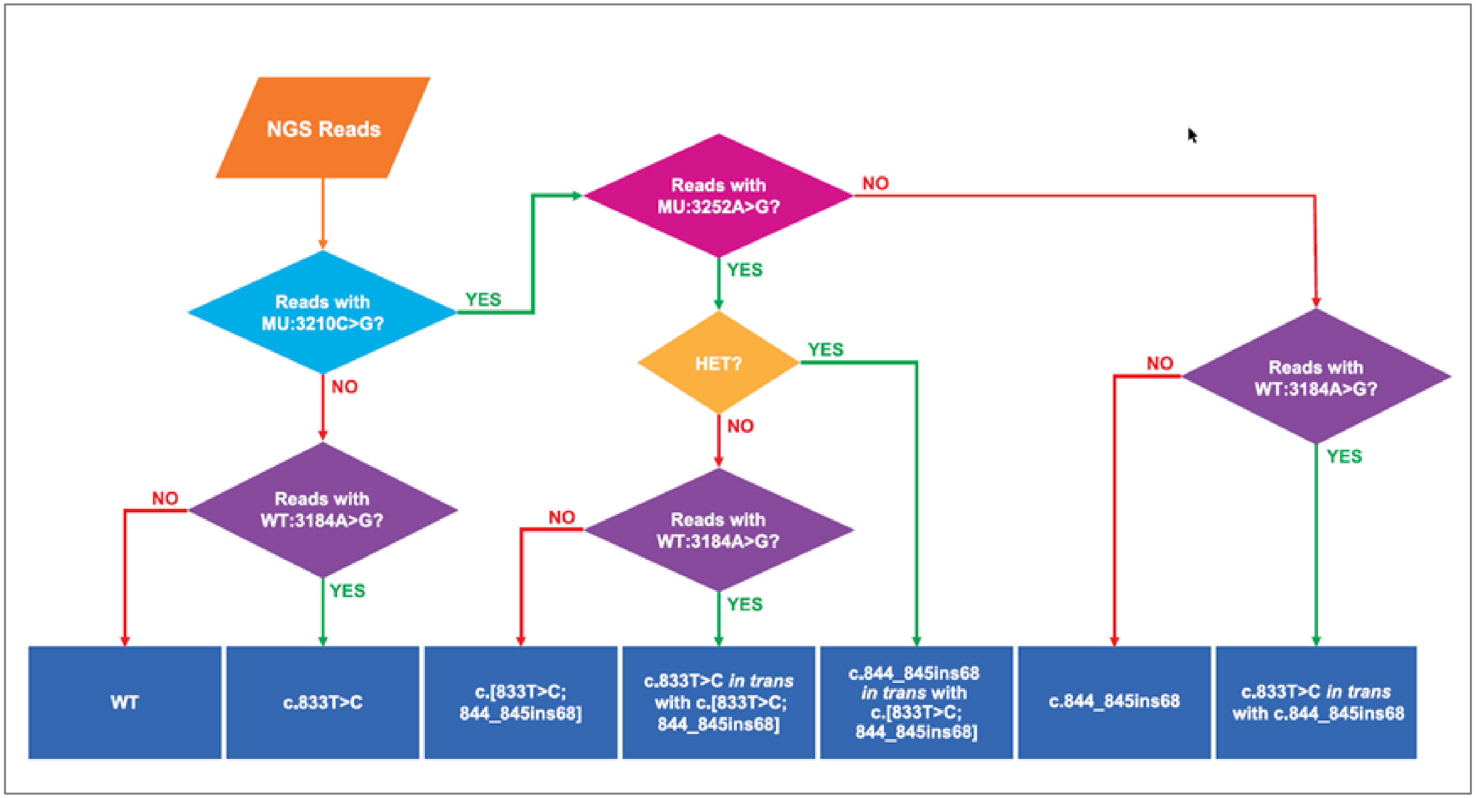

Supplementary Figure S1: Scaffolds alignment strategy for the *CBS* variants

A flowchart diagrams the *CBS* variants calling strategy for all possible combinations of c.833T>C and c.844_845ins68 using the custom scaffolds. Both detection of the informative base and the zygosity call are required for genotyping and phasing.

Supplementary Figure S2

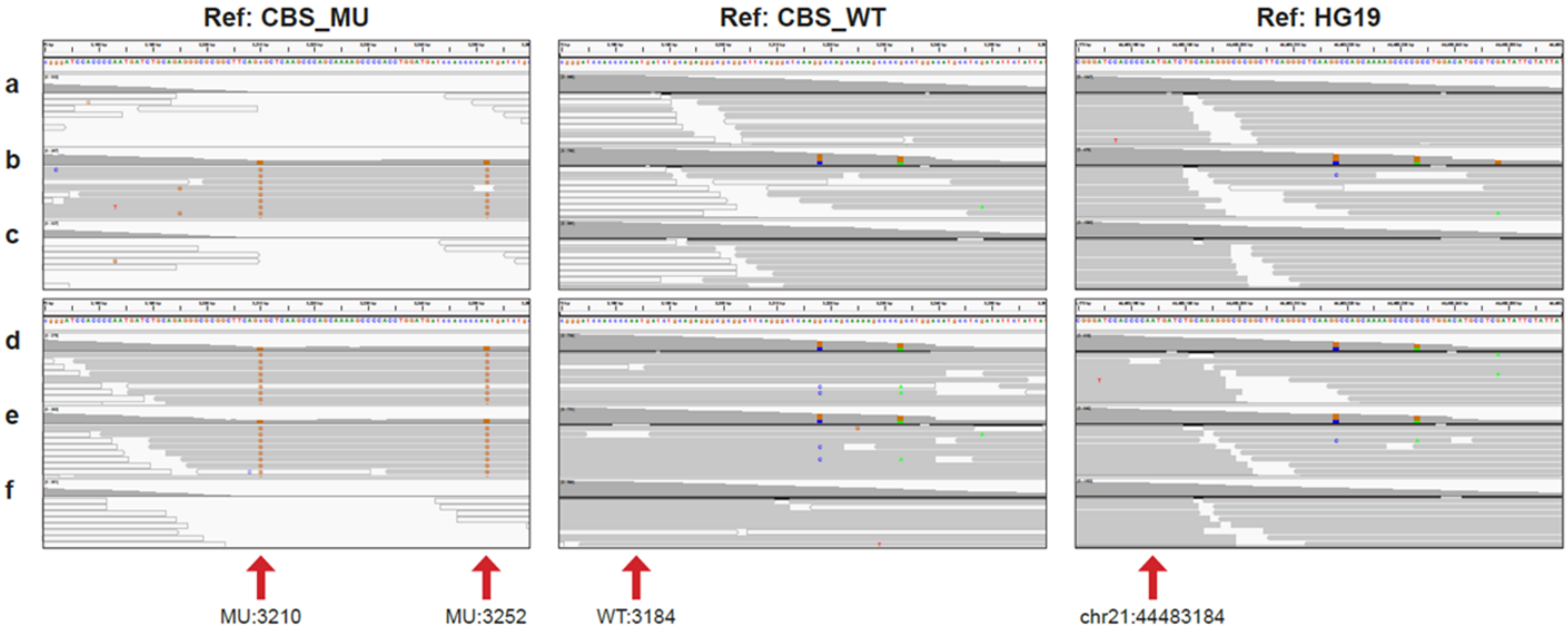

Supplementary Figure S2: Detection of c.[833T>C;844_845ins68] in two 1000G trios using custom scaffolds

Read alignments on Ref:CBS_MU, Ref:CBS_WT and Ref:HG19 for the CEPH trio (**a**) father, **(b)** mother, (**c**) daughter and YRI trio (**d**) father, **(e)** mother, **(f)** daughter are shown. Characteristic alignment profiles on Ref:CBS_MU indicate that the CEPH mother (**b**) and the YRI mother-daughter duo (**d,e**) carry the c.[833T>C;844_845ins68] complex variant. With Ref:HG19, the read alignment profiles are similar for all members of the CEPH and YRI trios (**a**-**f**). Arrows point to the informative bases that are used for variant calling. Two bases that differ between the 68bp insertion and HG19 reference sequence can be identified in the per-base track (green “A” and blue “C”). The third divergent base at the end of the insertion sequence is not visible in the alignments due to soft-clipping.

Supplementary Figure S3

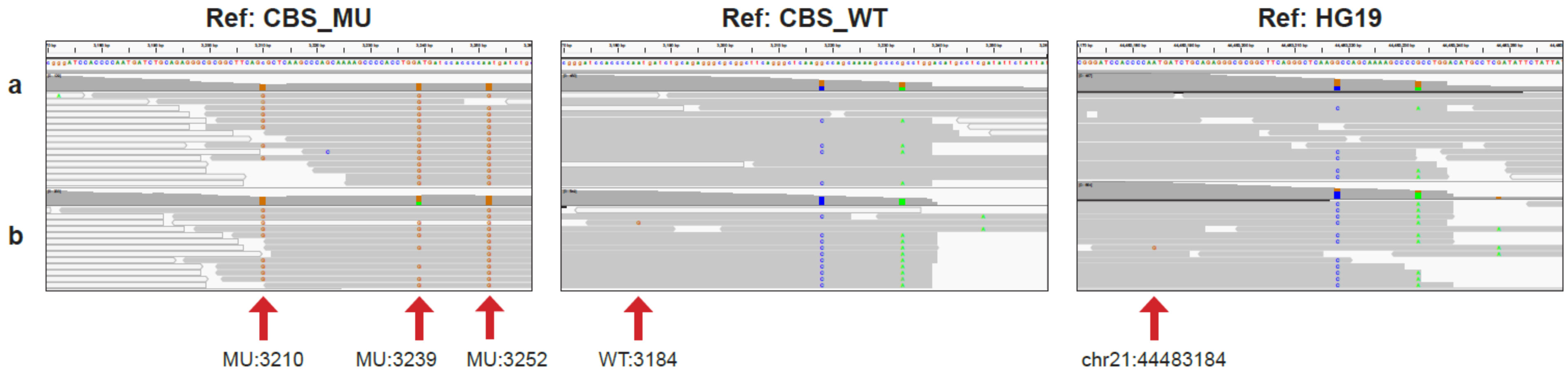

Supplementary Figure S3: Detection of the rare 68bp insertion in clinical samples via the CBS_MU scaffold

Shown are representative examples of (**a**) a sample that is heterozygous for a rare allele of the 68bp insertion, and (**b**) a sample that is a compound heterozygote for the rare and common 68bp insertion alleles that are both in *cis* with c.833T>C. Note that reads with the rare 68bp insertion allele are detected by an additional readout at MU:3239 on the Ref:CBS_MU scaffold. Two bases that differ between the 68 bp insertion and the HG19 reference sequence can be identified in the per-base track with the Ref:CBS_WT and Ref:HG19 alignments (green "A" and blue "C"). Two other divergent bases in the rare 68bp insertion allele are soft-clipped and not visible in the alignments because they occur at the very end of the insertion sequence.
